# Frequency and Longitudinal Course of Motor Signs in Genetic Frontotemporal Dementia

**DOI:** 10.1212/WNL.0000000000200828

**Published:** 2022-09-06

**Authors:** Sonja Schönecker, Francisco J. Martinez-Murcia, Boris-Stephan Rauchmann, Nicolai Franzmeier, Catharina Prix, Elisabeth Wlasich, Sandra V. Loosli, Katja Bochmann, Juan-Manuel Gorriz Saez, Robert Laforce, Simon Ducharme, Maria Carmela Tartaglia, Elizabeth Finger, Alexandre de Mendonça, Isabel Santana, Raquel Sanchez-Valle, Fermin Moreno, Sandro Sorbi, Fabrizio Tagliavini, Barbara Borroni, Markus Otto, Matthis Synofzik, Daniela Galimberti, Rik Vandenberghe, John van Swieten, Christopher Butler, Alexander Gerhard, Caroline Graff, Adrian Danek, Jonathan D. Rohrer, Mario Masellis, James Rowe, Johannes Levin, on behalf of The Genetic Frontotemporal Dementia Initiative (GENFI)

**Affiliations:** From the Department of Neurology (S. Schönecker, C.P., E.W., S.V.L., A.D., J.L.), Ludwig-Maximilians-Universität München, Germany; Department of Signal Theory Networking and Communications (F.J.M.-M., J.-M.G.S.), Andalusian Research Institute in Data Science and Computational Intelligence (DaSCI), University of Granada, Spain; Institute for Clinical Radiology (B.-S.R.), Institute for Stroke and Dementia Research (N.F.), and Institute of Neuroradiology (K.B.), Ludwig-Maximilians-Universität München, Germany; Département des Sciences Neurologiques (R.L.), Clinique Interdisciplinaire de Mémoire (CIME); McConnell Brain Imaging Centre (S.D.), Montreal Neurological Institute, McGill University; Department of Psychiatry (S.D.), McGill University Health Centre, McGill University, Montreal, Quebec; Tanz Centre for Research in Neurodegenerative Diseases (M.C.T.), University of Toronto; Department of Clinical Neurological Sciences (E.F.), University of Western Ontario, London, Canada; Department of Neurology and Laboratory of Neurosciences (A.M.), Faculty of Medicine, University of Lisbon; Center for Neuroscience and Cell Biology (I.S.), Faculty of Medicine, Centro Hospitalar e Universitário de Coimbra; Center for Neuroscience and Cell Biology (I.S.), Faculty of Medicine, University of Coimbra, Portugal; Alzheimer's Disease and Other Cognitive Disorders Unit (R.S.-V.), Neurology Service, Hospital Clinic, Institut d'Investigacions Biomediques August Pi I Sunyer; Institut d'Investigació Biomèdica August Pi I Sunyer (R.S.-V.), Barcelona; Department of Neurology (F.M.), Donostio University Hospital, San Sebastian; Neuroscience Area (F.M.), Biodonostia Health Research Institute, San Sebastian, Gipuzkoa, Spain; Department of Neuroscience, Psychology, Drug Research and Child Health (S. Sorbi), University of Florence; IRCCS Fondazione Don Carlo Gnocchi (S. Sorbi), Florence; Fondazione Istituto di Ricovero e Cura a Carattere Scientifico Istituto Neurologica Carlo Besta (F.T.), Milano; Centre for Neurodegenerative Disorders (B.B.), Neurology Unit, Department of Clinical and Experimental Sciences, University of Brescia, Italy; Department of Neurology (M.O.), University Hospital Ulm; Department of Neurology (M.O.), Martin-Luther-University Halle-Wittenberg, Germany Department of Neurodegenerative Diseases (M.S.), Hertie-Institute for Clinical Brain Research and Center of Neurology, University of Tübingen; Center for Neurodegenerative Diseases (M.S.), Tübingen, Germany; Fondazione IRCCS Ospediale Policlinico (D.G.), Milan; Centro Dino Ferrari (D.G.), University of Milan, Italy; Leuven Brain Institute (LBI) (R.V.), KU Leuven; Laboratory for Cognitive Neurology (R.V.), Department of Neurosciences, KU Leuven; Neurology Department (R.V.), UZ Leuven, Belgium; Department of Neurology (J.S.), Erasmus Medical Centre, Rotterdam, the Netherlands; Nuffield Department of Clinical Neurosciences (C.B.), Medical Sciences Division, University of Oxford; Department of Brain Sciences (C.B.), Imperial College London; Wolfson Molecular Imaging Centre (A.G.), Faculty of Medicine, Biology and Health, University of Manchester, United Kingdom; Departments of Geriatric Medicine and Nuclear Medicine (A.G.), Essen University Hospital, Germany; Swedish FTD Initiative (C.G.), Stockholm; Division of Neurogeriatrics (C.G.), Centre for Alzheimer Research, Department of Neurobiology, Care Sciences and Society, Karolinska Institutet Solna; Unit for Hereditary Dementias (C.G.), Theme Aging, Karolinska University Hospital, Stockholm, Sweden; Dementia Research Centre (J.D.R.), University College London, United Kingdom; Hurvitz Brain Sciences Program (M.M.), Sunnybrook Research Institute, University of Toronto; Division of Neurology (M.M.), Department of Medicine, University of Toronto; Cognitive and Movement Disorders Clinic (M.M.), Sunnybrook Health Sciences Centre, Toronto, Ontario, Canada; Cognition and Brain Sciences Unit (J.R.), Medical Research Council; Department of Clinical Neurosciences (J.R.), University of Cambridge; Cambridge University Hospitals NHS Trust (J.R.), United Kingdom; German Center for Neurodegenerative Diseases (DZNE) (J.L.); Munich Cluster for Systems Neurology (SyNergy) (J.L.); and European Reference Network for Rare Neurological Diseases (ERN-RND) (J.L.), Munich, Germany.

## Abstract

**Background and Objectives:**

Frontotemporal dementia (FTD) is a highly heritable disorder. The majority of genetic cases are caused by autosomal dominant pathogenic variants in the chromosome 9 open reading frame 72 (*c9orf72*), progranulin (*GRN*), and microtubule-associated protein tau (*MAPT*) gene. As motor disorders are increasingly recognized as part of the clinical spectrum, the current study aimed to describe motor phenotypes caused by genetic FTD, quantify their temporal association, and investigate their regional association with brain atrophy.

**Methods:**

We analyzed baseline visit data of known carriers of a pathogenic variant in the *c9orf72*, *GRN*, or *MAPT* gene from the Genetic Frontotemporal Dementia Initiative cohort study. Principal component analysis with varimax rotation was performed to identify motor sign clusters that were compared with respect to frequency and severity between groups. Associations with cross-sectional atrophy patterns were determined using voxel-wise regression. We applied linear mixed effects models to assess whether groups differed in the association between motor signs and estimated time to symptom onset.

**Results:**

A total of 322 pathogenic variant carriers were included in the analysis: 122 *c9orf72* (79 presymptomatic), 143 *GRN* (112 presymptomatic), and 57 *MAPT* (43 presymptomatic) pathogenic variant carriers. Principal component analysis revealed 5 motor clusters, which we call progressive supranuclear palsy (PSP)-like, bulbar amyotrophic lateral sclerosis (ALS)-like, mixed/ALS-like, Parkinson disease (PD) like, and corticobasal syndrome–like motor phenotypes. There was no significant group difference in the frequency of signs of different motor phenotypes. However, mixed/ALS-like motor signs were most frequent, followed by PD-like motor signs. Although the PSP-like phenotype was associated with mesencephalic atrophy, the mixed/ALS-like phenotype was associated with motor cortex and corticospinal tract atrophy. The PD-like phenotype was associated with widespread cortical and subcortical atrophy. Estimated time to onset, genetic group and their interaction influenced motor signs. In *c9orf72* pathogenic variant carriers, motor signs could be detected up to 25 years before expected symptom onset.

**Discussion:**

These results indicate the presence of multiple natural clusters of motor signs in genetic FTD, each correlated with specific atrophy patterns. Their motor severity depends on time and the affected gene. These clinicogenetic associations can guide diagnostic evaluations and the design of clinical trials for new disease-modifying and preventive treatments.

Frontotemporal dementia (FTD) refers to a heterogeneous group of neurodegenerative disorders. The associated clinical syndromes classically affect personality and social behavior or language.^1^ They are a common cause of early-onset dementia^2^ and are highly heritable.^3,4^ The majority of genetic cases are caused by pathogenic variants in 1 of 3 genes: chromosome 9 open reading frame 72 (*c9orf72*),^5^ progranulin (*GRN*),^6^ and microtubule-associated protein tau (*MAPT*).^7^ *C9orf72* pathogenic variants are most common.

Because of the clinical heterogeneity, a precise knowledge of clinical presentations correlated with genetic subgroups is essential to guide diagnostic workup and assist decisions regarding genetic testing. It will also become increasingly important as disease-modifying drug trials are underway in each of the 3 genetic FTD groups.^8-10^

Patients can also present with a wide range of motor signs, including those commonly associated with amyotrophic lateral sclerosis (ALS),^11^ Parkinson disease (PD), progressive supranuclear palsy (PSP), or corticobasal syndrome (CBS).^12,13^ We propose that the anatomic distribution of pathologic brain changes determines the clinical phenotype. This distribution can be defined in terms of brain regions^14^ or functionally in terms of degeneration of the first and second motor neuron and basal ganglia.^15^ The identification of motor structure-function relationships in sporadic FTD is hindered by uncertainty of the molecular pathology. This challenge is addressed by the analysis of genetic FTD.

Although there is a wide literature covering behavioral and linguistic features in FTD, detailed phenotypic characterization of motor disorders is mostly in sporadic cases^14^ or in the form of case reports or case series.^16^ Longitudinal data on motor phenotypes are lacking. We aimed to describe motor phenotypes in genetic FTD, from the Genetic Frontotemporal dementia Initiative (GENFI). We examined motor sign occurrence in the course of the disease, including the presymptomatic phase, and tested whether structural brain changes are associated with particular motor phenotypes.

## Methods

### Standard Protocol Approvals, Registrations, and Patient Consents

The study was performed according to the Declaration of Helsinki (1991). Ethical approval for conduction of the study has been obtained at the coordinating site at University College London and all participating centers. Written informed consent was obtained from every participant.

### Participants

To assess motor findings in genetic FTD, we used Data Freeze 3 from the GENFI multicenter cohort study, gathered between January 30, 2012, and January 31, 2017. The GENFI consists of research centers across Europe and Canada (genfi.org.uk/) and enrolls participants who are known carriers of a pathogenic variant in *c9orf72*, *GRN*, or *MAPT* or are at risk of carrying a pathogenic variant because a first-degree relative was a known carrier. A pathogenic *c9orf72* expansion was defined as more than 30 repeats. Participants were genotyped at their local site. All eligible and interested participants were enrolled. A total of 322 pathogenic variant carriers, including 122 *c9orf72* (79 presymptomatic), 143 *GRN* (112 presymptomatic), and 57 *MAPT* pathogenic variant carriers (43 presymptomatic) were included in the analysis. Baseline visit data were used.

Participants underwent a standardized clinical assessment consisting of medical history, family history, and physical examination. Participants not yet demonstrating clear evidence of progressive cognitive, behavioral, or motor symptoms were classified as presymptomatic. Estimated years to symptom onset (EYO) was defined as the difference between the participants' current age and the mean age at onset within the participants' family.^17^

### Assessment of Motor Impairment

The presence and severity of the following signs was assessed: supranuclear gaze palsy, impaired eyelid function, facial weakness, bulbar palsy, pseudobulbar palsy, neck weakness, neck rigidity, respiratory muscle weakness, myoclonus, rest tremor, postural tremor, dystonia, chorea, bradykinesia, rigidity, limb apraxia, alien limb phenomenon, cortical sensory loss, limb fasciculations, spasticity, limb weakness, hyperreflexia, ataxia, arising from chair, sitting down, and postural instability. Severity of signs was scored as follows: score 0 = no impairment, score 0.5 = very mild impairment, score 1 = mild impairment, score 2 = moderate impairment, and score 3 = severe impairment (eTable 1, links.lww.com/WNL/C158). For motor signs affecting different limbs, the score of the most severely affected limb was used. To assess limb motor asymmetry, laterality indices (LIs) defined as the difference of left and right were calculated. For motor signs scoring 4 extremities, the mean of side differences was calculated. To assess overall asymmetry, the amount of the sum of all LIs was used.

### MRI Acquisition and Analysis

MRI data were available in 286/322 patients. MRIs were acquired on a 3 T scanner with a 1.1 mm isotropic resolution (GE, Philips, Siemens Prisma, Siemens Skyra, Siemens Trio). Acquisition protocols were synchronized across scanners and sites to achieve the best possible match.

Voxel-based morphometry was performed using the Statistical Parametric Mapping toolbox (SPM12)^18^ in MATLAB (MathWorks, Natick, MA). Images were segmented into probability maps of gray matter, white matter, and CSF, nonlinearly transformed using DARTEL^19^ to create a study specific template for white and gray matter and normalized to the Montreal Neurological Institute space with Jacobian modulation. Spatial smoothing was applied using a full width at half maximum 6-mm Gaussian kernel. An estimate of total intracranial volume for each subject was computed by summing the 3 tissue class volumes.^20^

### Statistical Analysis

Data were analyzed using IBM SPSS Statistics for Windows (version 25.0; IBM Corp., Armonk, NY). Nondichotomized mean scores of demographic data were compared via the Kruskal-Wallis test and post hoc Bonferroni-corrected Mann-Whitney test. Chi-square analysis was used to check for significant differences in sex. Standard statistical significance level was set at *p* < 0.05.

To identify groups of similar clinical variables, our set of motor scores as well as overall LI were subjected to a principal component analysis (PCA) with varimax rotation. Variables with factor loadings below 0.4 were eliminated from the analysis and the PCA run anew. Components were labeled post hoc according to the pattern of signs. No a priori assumptions regarding the clustering of motor signs were applied. To visualize the similarity of variables assigned to a specific component during PCA, multidimensional scaling (MDS) was performed. Furthermore, to visualize possible gene clustering between phenotype clusters, a between-cases MDS was performed. For each group, the variance in each dimension was calculated, and a Levene test was performed to assess possible inequality of variances.

To test for differences of motor signs depending on the affected gene, we calculated for each participant a sum score from the variables of each component. As overall LI has a different scale than the other variables, it was analyzed separately. Sum scores were compared via Kruskal-Wallis and post hoc Bonferroni-corrected Mann-Whitney tests between groups.

To assess the proportion of the predominant phenotype of patients with motor signs depending on the affected gene, cases were assigned to the component with the highest PCA-based sum score. In addition, the frequency of signs of different components was determined for each group. Chi-square analysis was used to check for significant differences in frequency of signs.

We assessed for each component the association between the sum scores and the patterns of atrophy using linear regression models. Data of patients with a sum score of 0 were excluded. The estimate of total intracranial volume was included as a covariate. Probability maps of gray matter and white matter were analyzed separately. T-maps were merged for visualization purposes. Images of the association between the sum scores of component 5 and cross-sectional atrophy patterns were partly flipped according to the expected atrophy pattern. The hemisphere with the expected atrophy (based on lateralization of motor symptoms) was arranged to the left. Absolute threshold masking was set at 0.1 to prevent interference by nonbrain voxels (*p* < 0.001, cluster threshold k = 20 voxel).

In addition, we applied linear mixed-effects (LME) models^21^ to assess differences between genetic groups in function of the calculated sum scores. Via LME we performed a modeling of the predictor variables as a linear model combining fixed and random effects; the former accounting for known sources of variation such as groups or time, the later accounting for the variance contribution of clusters in the data and correlations within members of each cluster.

We tested several models including random intercepts per family and site.^17^ Fixed effect variables included EYO, genetic group, and sex, along with interactions between genetic group and EYO. Nonlinear time dependence was expected, so a second-order contribution of EYO, including an interaction with genetic group, was added to the model. Higher-order contributions and logarithmic transformations were tested with no significant improvement of the model.

We applied a type II Wald χ^2^ test to the model, to estimate the relationship between the fixed variables and the sum scores. Afterward, the 3-way empirical significance was estimated from a Monte-Carlo sampling of the models for each sum score^22^ every 5 years in the EYO range from −25 to +10 to identify each sign's degree of differentiation. As indicator of the point in time at which motor signs of each component and genetic group start to increase, the time at which the lower 95% CI crosses zero on the x-axis was used. These analyses were performed using R 3.6.3.

### Data Availability

Data will be shared according to the GENFI data sharing agreement, after review by the GENFI data access committee with final approval granted by the GENFI steering committee.

## Results

### Demographics

Demographics of the study sample are provided in Table 1. *MAPT* pathogenic variant carriers were younger compared with the other groups. The proportion of presymptomatic participants was lower in *c9orf72* compared with *GRN* pathogenic variant carriers. Groups did not differ in terms of education, sex, and EYO.^17,23^

**Table 1:**
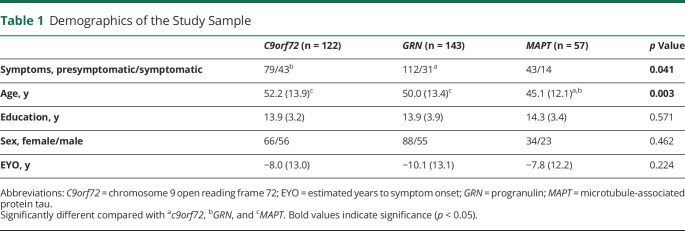
Demographics of the Study Sample

### PCA and Multidimensional Scaling

Both the Bartlett test (χ^2^ (351) 7,662.23, *p* < 0.001) and the Kaiser-Meyer-Olkin Measure of Sampling Adequacy (KMO = 0.766) indicated that variables were suitable for PCA with varimax rotation, which revealed the presence of 7 components with eigenvalues above 1. As 2 of these components contained only 2 variables, these were excluded from the analysis, leaving a 5-component solution explaining 67.3% of variance. The motor sign chorea was excluded as its factor loadings were below 0.4.

The variables group in the components as follows (details in Table 2):

1. Neck rigidity, impaired eyelid function, supranuclear gaze palsy, dystonia, pseudobulbar palsy, and ataxia: we call this the PSP-like motor phenotype (PSP-MP).
2. Respiratory muscle weakness, neck weakness, bulbar palsy, facial weakness, and myoclonus: we call this the bulbar ALS-like motor phenotype (bulbar ALS-MP).
3. Spasticity, limb weakness, limb fasciculations, arising from chair, bradykinesia, sitting down, postural instability, and hyperreflexia: we call this the mixed/ALS-like motor phenotype (mixed/ALS-MP), as this phenotype contains both nonspecific motor signs like bradykinesia and typical ALS features like spasticity, limb weakness, fasciculations, and hyperreflexia.
4. Rest tremor, postural tremor, overall LI, and rigidity: we call this the PD-like motor phenotype (PD-MP).
5. Cortical sensory loss, limb apraxia, and alien limb phenomenon: we call this the CBS-like motor phenotype (CBS-MP).

**Table 2:**
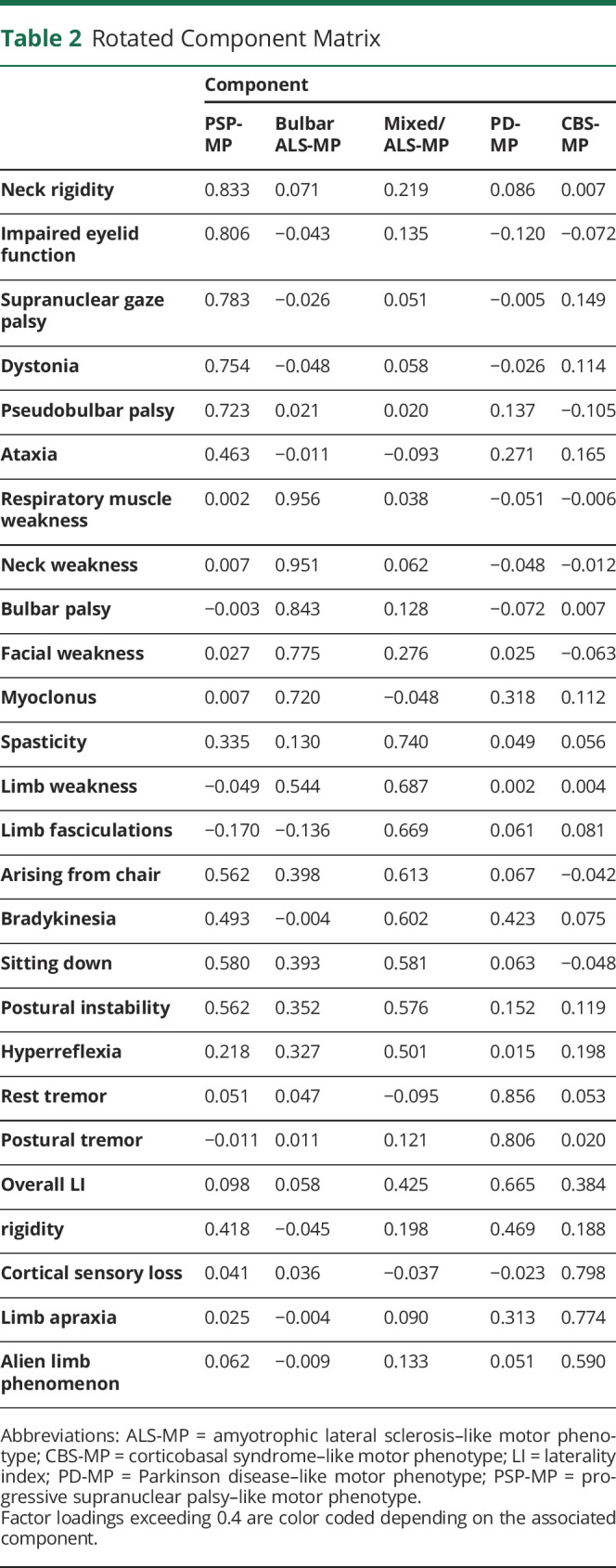
Rotated Component Matrix

**Figure 1:**
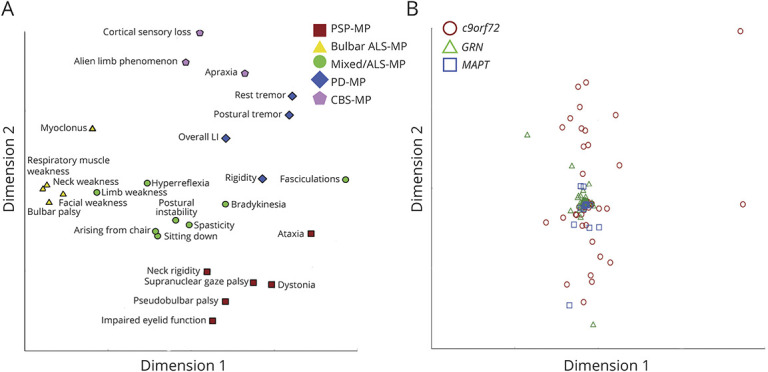
Multidimensional Scaling of Motor Signs and Genetic Cases, Respectively (A) Two-dimensional spatial representation based on the similarity of clinical variables as revealed by MDS. Variables that have been assigned to a specific motor phenotype by PCA are color coded. (B) Two-dimensional spatial representation based on the similarity of cases as revealed by MDS. Cases are color coded according to their affected gene. ALS = amyotrophic lateral sclerosis; *c9orf72* = chromosome 9 open reading frame 72; CBS = corticobasal syndrome; *GRN* = progranulin; *MAPT* = microtubule-associated protein tau; MDS = multidimensional scaling; MP = motor phenotype; PCA = principal component analysis; PD = Parkinson disease; PSP = progressive supranuclear palsy.

Of note, high scores on a certain component do not make a specific diagnosis, the names given are but a simplified label for a cluster of signs. MDS confirmed the grouping of variables as reasonable (normalized raw stress 0.040) (Figure 1A). In addition, a between-cases MDS (normalized raw stress 0.002) was performed (Figure 1B). The Levene test detected significant inequality of variances in dimension 1 between groups (*p* = 0.039) with highest variances in *c9orf72* pathogenic variant carriers. No significant group differences were detected in dimension 2.

### Severity of Motor Signs

**Figure 2:**
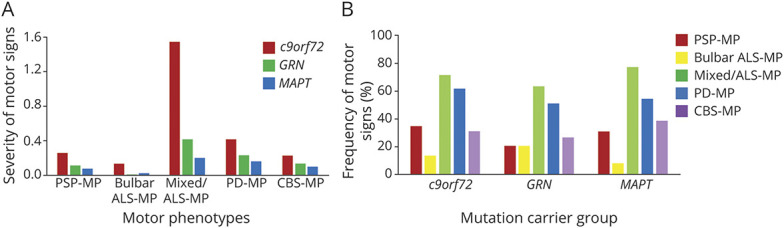
Severity and Frequency of Motor Signs (A) Comparison of the severity of motor signs as defined by the sum scores of the individual motor phenotypes according to the affected gene. (B) Comparison of the frequency of motor signs between pathogenic variant carriers showing motor signs. Patients may present motor signs of different phenotypes; therefore, the sum of frequencies does not add up to 100%. ALS = amyotrophic lateral sclerosis; *c9orf72* = chromosome 9 open reading frame 72; CBS = corticobasal syndrome; *GRN* = progranulin; *MAPT* = microtubule-associated protein tau; MP = motor phenotype; PD = Parkinson disease; PSP = progressive supranuclear palsy.

The Kruskal-Wallis test detected significant group differences of sum scores of the bulbar ALS-MP, mixed/ALS-MP, and PD-MP with highest sum scores in *c9orf72* pathogenic variant carriers (Figure 2A). Sum scores of the mixed/ALS-MP and PD-MP were lowest in *MAPT* pathogenic variant carriers, whereas sum scores of the bulbar ALS-MP were lowest in *GRN* pathogenic variant carriers. Sum scores of the PSP-MP and CBS-MP were highest in *c9orf72* and lowest in *MAPT* pathogenic variant carriers; however, statistical significance was not reached. As presymptomatic participants were largely normal on their clinical examination, differences of sum scores between groups at baseline examination were mainly driven by symptomatic participants.

### Frequency of Motor Signs

When looking at the group of pathogenic variant carriers showing motor signs, no significant group differences could be detected regarding the frequency of signs of different motor phenotypes (Figure 2B). This was similar when looking at the whole group (eFigure 1, links.lww.com/WNL/C158), χ^2^ analysis detected only significant group differences regarding the frequency of signs of the bulbar ALS-MP with highest frequency of signs in *c9orf72* pathogenic variant carriers.

Signs of the mixed/ALS-MP were most frequent across groups (63.3%–76.9%), followed by signs of the PD-MP (51.0%–61.5%). Signs of the CBS-MP (26.5% and 38.5%, respectively) were slightly more frequent than signs of the PSP-MP (20.4% and 30.8%, respectively) in *GRN* and *MAPT* pathogenic variant carriers. In contrast, in *c9orf72* pathogenic variant carriers, signs of the PSP-MP (34.7%) occurred more frequently than signs of the CBS-MP (30.8%). The least common signs were those of the bulbar ALS-MP (7.7%–13.5%). This was the case in all genetic groups, regardless of whether the cohort of patients showing motor signs or the whole cohort was analyzed.

### Predominance Phenotype

**Figure 3:**
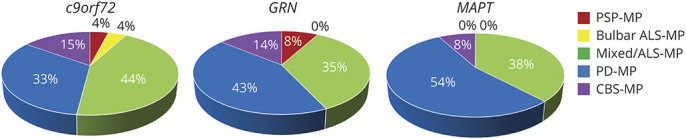
Proportion of the Dominant Clinical Phenotype of Patients With Motor Signs Depending on the Affected Gene Cases were assigned to the component with the highest PCA-based sum score. As patients may present motor signs of other motor phenotypes in addition to the signs of the predominating motor phenotype, this figure is not congruent with Figure 2B. ALS = amyotrophic lateral sclerosis; *c9orf72* = chromosome 9 open reading frame 72; CBS = corticobasal syndrome; *GRN* = progranulin; *MAPT* = microtubule-associated protein tau; MP = motor phenotype; PCA = principal component analysis; PD = Parkinson disease; PSP = progressive supranuclear palsy.

In *c9orf72* pathogenic variant carriers, the most frequent predominant phenotype was the mixed/ALS-MP (44%), followed by signs of the PD-MP (33%) (Figure 3), whereas this was the other way round in *GRN* and *MAPT* pathogenic variant carriers (PD-MP: 43% and 54%, respectively; mixed/ALS-MP: 35% and 38%, respectively). Although 21.7% of *c9orf72* pathogenic variant carriers with a predominant mixed/ALS-MP had confirmed motor neuron disease, in none of the patients with a *GRN* or *MAPT* pathogenic variant, motor neuron disease was diagnosed. The third most common predominant phenotype was the CBS-MP in all genetic groups, which was equally frequent in *c9orf72* and *GRN* (≈15%) and slightly less frequent in *MAPT* pathogenic variant carriers (8%). In *c9orf72* pathogenic variant carriers, the PSP-MP (4%) was slightly more frequent than the bulbar ALS-MP (2%). All *c9orf72* pathogenic variant carriers with a predominant bulbar ALS-MP had confirmed motor neuron disease. None of the *GRN* pathogenic variant carriers showed a predominant bulbar ALS-MP, and no *MAPT* pathogenic variant carrier showed a predominant PSP-MP or mixed/ALS-MP.

### Atrophy Patterns

**Figure 4:**
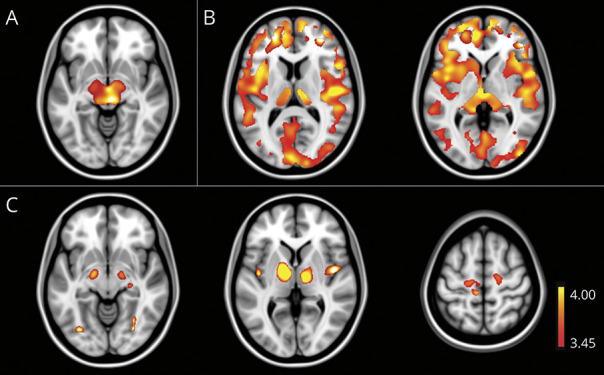
Correlation of Sum Scores of Motor Phenotypes With Cerebral Atrophy Using Linear Regression Models T-maps from the analysis of gray and white matter were merged for visualization purposes. (A) PSP-like motor phenotype, arising from *c9orf72*, *GRN*, and *MAPT* pathogenic variants, not progressive supranuclear palsy pathology. (B) PD-like motor phenotype, arising from *c9orf72*, *GRN*, and *MAPT* pathogenic variants, not PD. (C) Mixed/ALS-like motor phenotype, arising from *c9orf72*, *GRN*, and *MAPT* pathogenic variants. ALS = amyotrophic lateral sclerosis; *c9orf72* = chromosome 9 open reading frame 72; CBS = corticobasal syndrome; *GRN* = progranulin; *MAPT* = microtubule-associated protein tau; PD = Parkinson disease; PSP = progressive supranuclear palsy.

Voxel-wise regression revealed sum scores of the PSP-MP to be highly correlated with mesencephalic atrophy (Figure 4, eFigure 3, eTable 2, links.lww.com/WNL/C158). Atrophy clusters correlating with sum scores of the bulbar ALS-MP were rather small and distributed over all lobes with a focus on the frontal and temporal lobe. For the mixed/ALS-MP, the clusters of white matter atrophy were mainly located in extranuclear and brain stem white matter as well as in subcortical white matter of the medial frontal and precentral gyrus. Clusters of gray matter atrophy were located in the precentral, medial frontal, and superior frontal gyrus (Figure 4C). In addition, clusters of gray matter atrophy could be detected in both cerebellar tonsils, the left declive, insula and posterior cingulate. Sum scores of the PD-MP showed a high correlation with diffuse cerebral and cerebellar cortical and subcortical atrophy (Figure 4B). Only small atrophy clusters correlating with the sum scores of the CBS-MP mainly located in the temporal, occipital, and parietal lobes could be detected.

### LME Models

**Figure 5:**
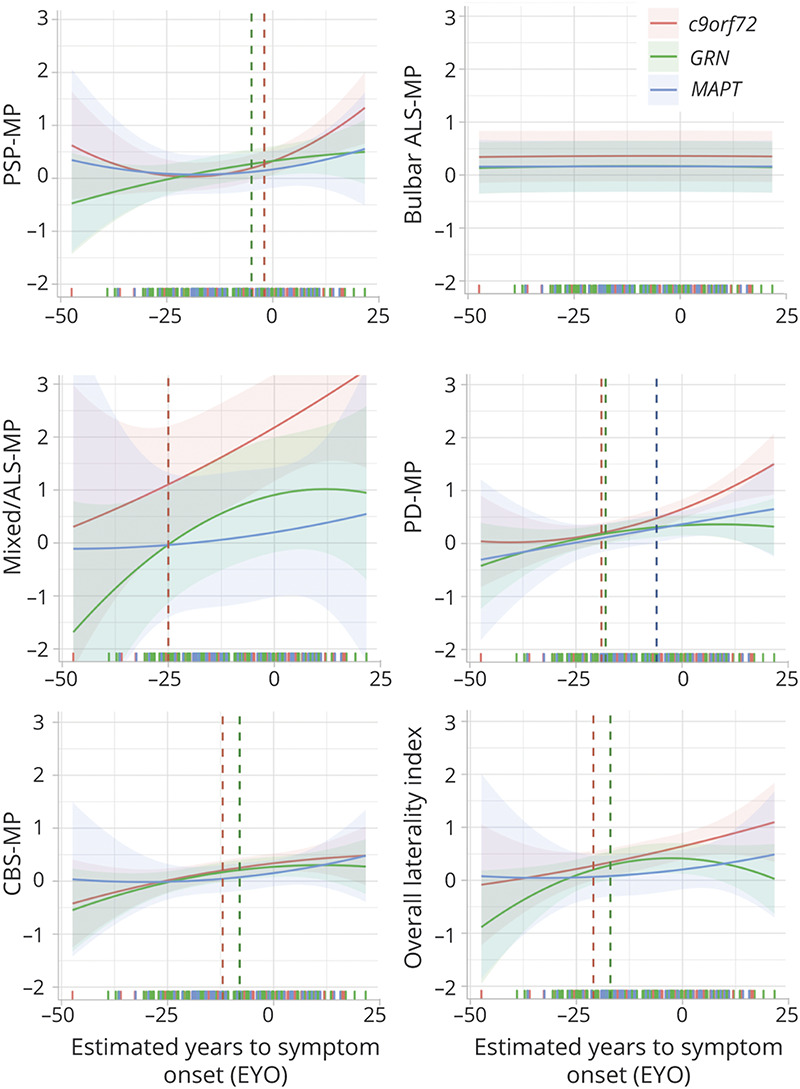
Calculated Sum Scores and Overall Laterality Index (With 95% CIs), Respectively, vs Estimated Years to Symptom Onset An early increase of motor signs, up to 25 years before the expected symptom onset, could be detected in *c9orf72* pathogenic variant carriers. In *MAPT* pathogenic variant carriers, motor signs occurred latest. The point in time at which the lower 95% CI of the model crosses the x-axis is marked by a vertical bar in the respective color for each group. Although the severity of motor signs remained highest in *c9orf72* pathogenic variant carriers over time, severity of motor signs of *GRN* and *MAPT* pathogenic variant carriers progressively converged. Individual data points are not plotted to prevent disclosure of genetic status. However, the time of the examination is marked on the x-axis by a colored dash. ALS = amyotrophic lateral sclerosis; *c9orf72* = chromosome 9 open reading frame 72; CBS = corticobasal syndrome; *GRN* = progranulin; *MAPT* = microtubule-associated protein tau; MP = motor phenotype; PD = Parkinson disease; PSP = progressive supranuclear palsy.

The visual distribution of sum scores and overall laterality over EYO is depicted in Figure 5. The Type II Wald χ^2^ test revealed a significant effect of EYO on the sum scores of the PSP-MP (*p* < 0.001), mixed/ALS-MP (*p* = 0.030), and PD-MP (*p* < 0.001) and a significant effect of genetic group on the sum scores of the mixed/ALS-MP (*p* < 0.001) and PD-MP (*p* = 0.016). The interaction of EYO and genetic group had a significant effect on the sum scores of the PD-MP (*p* = 0.027). None of the variables included in the model reached statistical significance when evaluating the sum scores of the bulbar ALS-MP and CBS-MP.

As a possible indicator of signs starting to emerge, we determined for each component and genetic group the point in time at which the lower 95% CI of the model crosses the x-axis. Although no clear onset of signs could be detected for the bulbar ALS-MP, in *c9orf72* and *GRN* pathogenic variant carriers, an increase of signs of the PSP-MP could be detected already shortly before estimated onset (Figure 5). In *c9orf72* pathogenic variant carriers, an increase of signs of the mixed/ALS-MP was detectable already 25 years before the estimated onset. Signs of the PD-MP started to increase more than 15 years before estimated onset in *c9orf72* and *GRN* pathogenic variant carriers and around 5 years before estimated onset in *MAPT* pathogenic variant carriers, whereas signs of the CBS-MP increased 10 years before estimated onset in *c9orf72* pathogenic variant carriers and more than 5 years before estimated onset in *GRN* pathogenic variant carriers.

Sum scores of the PD-MP were significantly higher in *c9orf72* compared with *GRN* and *MAPT* pathogenic variant carriers 5 years before estimated onset (Figure 5) and significantly higher in *MAPT* compared with *GRN* pathogenic variant carriers 15 years after estimated onset. Sum scores of the CBS-MP were significantly higher in *c9orf72* compared with *MAPT* pathogenic variant carriers 15 years before estimated onset and converged 15 years after estimated onset. Furthermore, they were significantly higher in *GRN* compared with *MAPT* pathogenic variant carriers 10 years before estimated onset and converged 10 years after estimated onset. The earliest point at which sum scores of the CBS-MP were significantly higher in *c9orf72* compared with *GRN* pathogenic variant carriers was 10 years after estimated onset. We noted no significant group differences of sum scores of the PSP-MP, bulbar ALS-MP, and mixed/ALS-MP over time.

## Discussion

We present a data-driven approach to demonstrate the phenotypic range of motor signs, their association with time to expected onset as well as with specific atrophy patterns in genetic FTD. PCA confirmed the presence of natural clusters of motor phenotypes, a PSP-like, a bulbar ALS-like, a mixed/ALS-like, a PD-like, and a CBS-like motor phenotype.

The prevalence of signs of these phenotypes in the overall cohort was similar across genetic groups. This is in line with a recent review and meta-analysis.^13^ However, in our cohort, signs of the mixed/ALS-MP were most frequent across groups, followed by signs of the PD-MP. The most common phenotype in *GRN* and *MAPT* pathogenic variant carriers was PD-MP, closely followed by mixed/ALS-MP: this was the other way round in *c9orf72* pathogenic variant carriers. This was to be expected in *c9orf72* pathogenic variant carriers but is rather unexpected in *GRN* and *MAPT* pathogenic variant carriers, as the occurrence of ALS-like signs has only rarely been described in these conditions.^4,24^ Of interest, this was only in part due to the nonspecific variables with high cross loadings on the PSP-MP and PD-MP contained in this phenotype cluster, as the frequency of the remaining signs of the third component was still 16.1% in *GRN* and 15.8% in *MAPT* pathogenic variant carriers, with the most frequent sign being hyperreflexia. However, typical ALS signs like limb weakness, fasciculations, and spasticity were present as well.

In previous reports, parkinsonism and Richardson syndrome have been described in association with MAPT pathogenic variants,^25-27^ but in our overall cohort, motor signs of a PSP-MP, PD-MP, and CBS-MP occurred most frequently in *c9orf72* followed by *GRN* pathogenic variant carriers. Intriguingly, no *MAPT* pathogenic variant carrier exhibited a predominant PSP-MP. This may be due to the small number of *MAPT* pathogenic variant carriers showing motor signs (n = 13). The fact that motor disorders and parkinsonism are typically brought into connection with a *MAPT* pathogenic variant may be caused by the earlier discovery of *MAPT* pathogenic variants in people with PSP-like phenotypes,^7^ 8 years earlier than *GRN*^6^ and 13 years earlier than *c9orf72*.^5^ Therefore, a higher number of papers reporting *MAPT* pathogenic variants may have skewed the perception of prevalence, leading to the impression that *MAPT* has a higher proportion of motor disorders.

The more frequent occurrence of signs of the CBS-MP in *c9orf72* compared with *GRN* pathogenic variant carriers is surprising as previous reports have described CBS to be most often associated with *GRN* pathogenic variants.^28-30^ This discrepancy may be due to the fact that most previous studies on motor disorders in genetic FTD have been case reports and case series that have focused on the predominance phenotype without describing accompanying low-grade signs. In fact, when looking solely at patients showing motor signs, *GRN* pathogenic variant carriers were similarly likely to show a predominant CBS-MP compared with *c9orf72* pathogenic variant carriers.

Signs of a bulbar ALS-MP were most frequent in *c9orf72* pathogenic variant carriers^31^ and could rarely be detected in *GRN* and *MAPT* pathogenic variant carriers. None of the *GRN* and *MAPT* pathogenic variant carriers showing motor signs exhibited a predominant bulbar ALS-MP. The presence of manifest bulbar signs therefore effectively excludes the presence of *GRN* and *MAPT* pathogenic variants.

Although the clinical phenotype is known to be highly heterogeneous across all pathogenic variants under investigation,^32^ the between-cases MDS performed demonstrates tightly overlapping phenotype clusters, albeit with higher variance in *c9orf72* pathogenic variant carriers and a more consistent syndrome for *GRN* and *MAPT*. This is reflected by the higher frequency and greater severity of signs across all phenotype clusters in *c9orf72* compared with *GRN* and *MAPT* pathogenic variant carriers. In agreement with the concept that the anatomy determines the phenotype,^15^ we were able to demonstrate strong clinicoanatomic correlations. This is reassuring about the validity of phenotype clusters defined by PCA. In agreement with previous studies on PSP-Richardson syndrome^33,34^ and PSP-like signs in sporadic FTD,^14^ the severity of the PSP-MP correlated with mesencephalic atrophy. The bulbar ALS-MP correlated with small areas of atrophy, mainly in the frontal and temporal lobe. This is consistent with previous studies reporting atrophy in frontotemporal regions, especially in patients additionally displaying behavioral or language signs,^35,36^ which was the case in all of our patients showing signs of the bulbar ALS-MP.

As expected, mixed/ALS-MP signs correlated with bilateral atrophy of the motor and premotor cortex and the corticospinal tracts including the internal capsule and brainstem.^35,37^ As in previous reports on the FTD-ALS continuum, clusters of atrophy in further frontotemporal regions^38^ and the cerebellum were also detectable.^39,40^

Surprisingly, there was only a small correlation of the PD-MP with basal ganglia atrophy but widespread cortical and subcortical correlates. Recent studies have demonstrated a similar widespread pattern of subtle bilateral cortical thinning involving frontal, parietal, temporal, and occipital lobes and extensive white matter damage already in early PD.^41-43^ However, our cases carry FTD pathogenic variants associated with TDP-43 and tau-pathology, not alpha-synucleinopathy. Dual pathology can occur but is very unlikely to be common across the GENFI cohort sufficient to cause the correlations with atrophy. The absence of basal ganglia atrophy may reflect the different underlying molecular pathology of PD-like MP in genetic FTD vs PD. The data suggest that mild PD-like signs in genetic FTD are rather due to diffuse cortical and subcortical atrophy than profound degeneration of the basal ganglia.

Even when flipping images according to the expected clinical atrophy pattern, only small atrophy clusters correlated with the CBS-MP. These were in the parietal, temporal, and occipital lobes. Although the clinical presentation of CBS is typically asymmetric, the variable overall LI was assigned to the PD-MP, not the CBS-MP by PCA, which explains why atrophy clusters correlating with the CBS-MP are not asymmetrically distributed. The fact that the factor loading of the variable overall LI on the CBS-MP was comparatively low may, however, be due to the small number of participants showing motor signs of the CBS-MP (n = 33).

Previous studies in CBS have demonstrated that atrophy patterns^44,45^ and cerebral glucose metabolism^46^ differ depending on the underlying pathology. Although in patients with corticobasal degeneration, the premotor cortex, supplemental motor area, and insula are typically affected, those with TDP-43 pathology exhibit pronounced frontotemporal atrophy, and patients with CBS with underlying Alzheimer pathology show more posterior atrophy, in parietal and temporal lobes. Although small, the atrophy clusters detected in our cohort seem to correspond to the atrophy detected in CBS caused by Alzheimer pathology. However, it is also possible that a differing distribution of pathology depending on the affected genes could have led to a mutual cancellation of atrophy patterns in our pooled analysis.

Previous studies in genetic FTD described changes in neuropsychological measures and structural imaging 5–10 years before expected onset.^17^ We show the emergence of motor signs up to 25 years before expected onset. Furthermore, our results demonstrate that severity of signs depends on the affected gene and that its effect varies over time. Mixed/ALS-MP, PD-MP, and CBS-MP signs occurred earliest in *c9orf72* pathogenic variant carriers, in agreement with the early detectable structural imaging findings^17,47^ and slow progress described in some *c9orf72* patients.^48-50^ In contrast, in *MAPT* pathogenic variant carriers that have been typically described in association with motor disorders, motor signs occurred later. Although the severity of signs remained highest in *c9orf72* pathogenic variant carriers, severity with *GRN* and *MAPT* pathogenic variant carriers converged over time.

As the majority of participants are alive, no valid conclusion on the influence of motor signs on overall survival can be drawn. However, an effect of motor signs, especially of the bulbar and mixed/ALS-MP, seems likely and should be investigated in future studies.

Besides the high number of patients with genetic FTD and the prospective evaluation of signs, the identification of natural clusters of motor signs by PCA represents a key strength of our study. Applying a data-driven approach allows for an objective analysis that does not follow classical clinical concepts and is not influenced by a priori assumptions.

A limitation of the current study that needs to be considered is the lack of a comparison to data from healthy controls. However, the primary aim was to compare motor disorders and their development over time between the genetic groups under investigation. We analyzed only cross-sectional differences between different genetic groups at different times from estimated onset. Whether the progression of signs, especially in the presymptomatic phase when subtle signs may be challenging to measure, is followed within individuals has to be shown in future longitudinal studies. Furthermore, a replication of phenotypes in another cohort would be of interest but is difficult to pursue due to the rarity of genetic FTD. Another limitation is the method used for estimation of estimated years to symptom onset. There is a significant correlation between an individual's age at onset and mean familial age at onset for MAPT pathogenic variants. This correlation is weak for *c9orf72* and *GRN*, such that EYO becomes a surrogate of age.

Keeping these limitations in mind, our data reveal the presence of natural clusters of motor signs in genetic FTD. Their severity increases over time and depends on the affected gene. The emergence of motor signs occurs early in the presymptomatic period, up to 25 years before estimated onset. Motor phenotypes have distinctive anatomic correlates. Given the heterogeneity of signs and symptoms and phenotypic overlap, these clinicogenetic associations of motor phenotypes in genetic FTD will help clinicians in their diagnostic workup, assist in decision making regarding genetic testing, and the design of preventive and disease-modifying treatments.

## Glossary

ALS: amyotrophic lateral sclerosis
*c9orf72*: chromosome 9 open reading frame 72
CBS: corticobasal syndrome
EYO: estimated years to symptom onset
FTD: frontotemporal dementia
GENFI: Genetic Frontotemporal dementia Initiative
*GRN*: progranulin
LI: laterality index
LME: linear mixed effect
*MAPT*: microtubule-associated protein tau
MDS: multidimensional scaling
MP: motor phenotype
PCA: principal component analysis
PD: Parkinson disease
PSP: progressive supranuclear palsy
